# Supplementary material for: Circulating blood eNAMPT drives the circadian rhythms in locomotor activity and energy expenditure
Source: Nat Commun. 2023 Apr 8;14:1994. doi: 10.1038/s41467-023-37517-6 (PMC10082796; doi:10.1038/s41467-023-37517-6)
Supplement: Supplementary file 1 — Supplementary Information [file 41467_2023_37517_MOESM1_ESM.pdf]

Supplementary Information

Circulating blood eNAMPT drives the circadian rhythms in locomotor activity and energy expenditure

Jae Woo Park[1], Eun Roh[2], Gil Myoung Kang[3], So Young Gil[3], Hyun Kyong Kim[3], Chan Hee Lee[4], Won Hee Jang[1], Se Eun Park[1], Sang Yun Moon[1], Seong Jun Kim[1], So Yeon Jeong[1], Chae Beom Park[1], Hyo Sun Lim[1], Yu Rim Oh[1], Han Na Jung[3,5], Obin Kwon[6], Byung Soo Youn[7], Gi Hoon Son[8], Se Hee Min[3,5], Min-Seon Kim[3,5]

* Correspondence and requests for materials should be addressed to M.S.K (mskim@amc.seoul.kr).

This PDF file includes:

Supplementary Figure 1-20

Supplementary Table 1-5

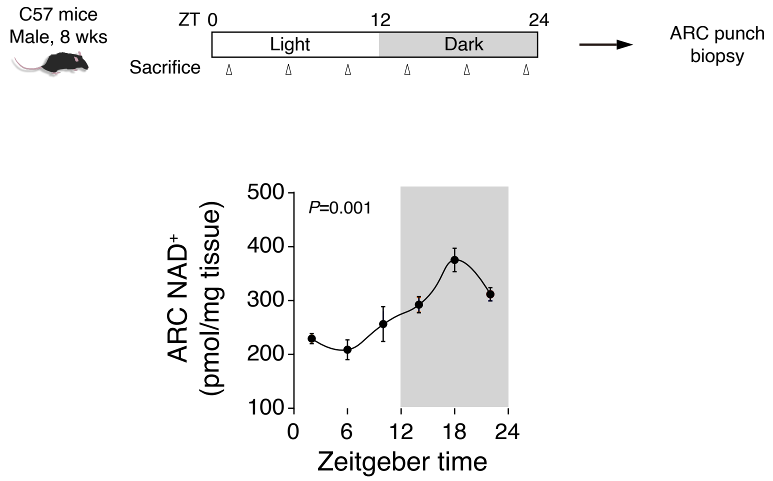

Supplementary Figure 1. Circadian variations in the NAD$^+$ levels of hypothalamic arcuate nucleus

Young male C57 mice were maintained under 12 h light-dark cycle and *ad libitum* fed condition. They were sacrificed every 4 h throughout a 24 h circadian cycle (n = 3). The hypothalamic arcuate nucleus (ARC) was collected using punch biopsy from hypothalmic slices. P value was determined using JTK-cycle algorithm. Two independent replicates were performed. ZT: zeitgeber time. Data are presented as mean ± SEM.

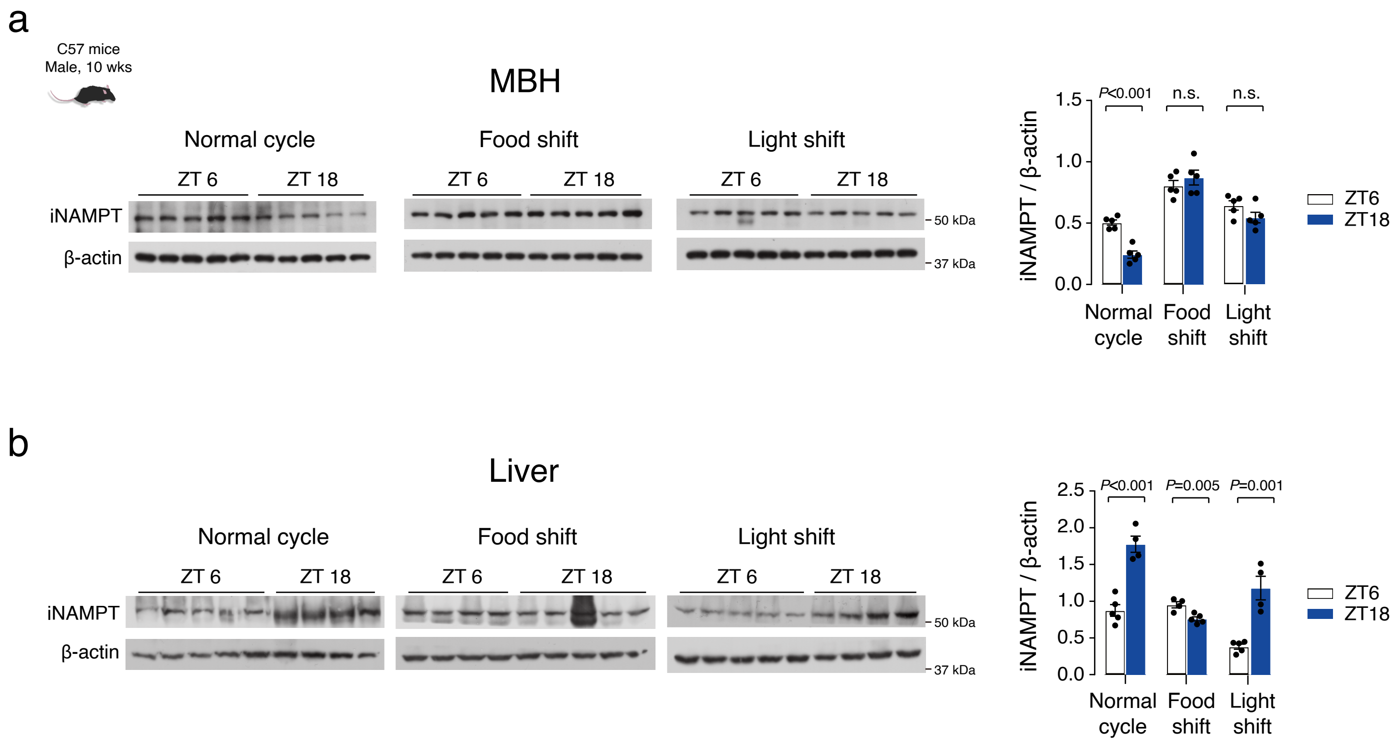

Supplementary Figure 2. Effects of light shift and food shift on the circadian oscillations of iNAMPT expression levels in the MBH and liver

a, **b** Mice were maintained on a normal light/dark cycle without food restriction (normal cycle), reverse light/dark cycle in *ad libitum* fed (light-shift), or allowed access to food only during the light period under a normal light/dark cycle (food-shift) for 14 days (Liver normal cycle ZT18, food shift ZT6 and light shift ZT18, n = 4; other groups, n = 5). Mice were sacrificed at 2 p.m. or 2 a.m. to collect the mediobasal hypothalamus (MBH) and liver. Unparied *t*-test (two-sided). Two independent replicates were performed. n.s.: not significant. All data are presented as mean ± SEM.

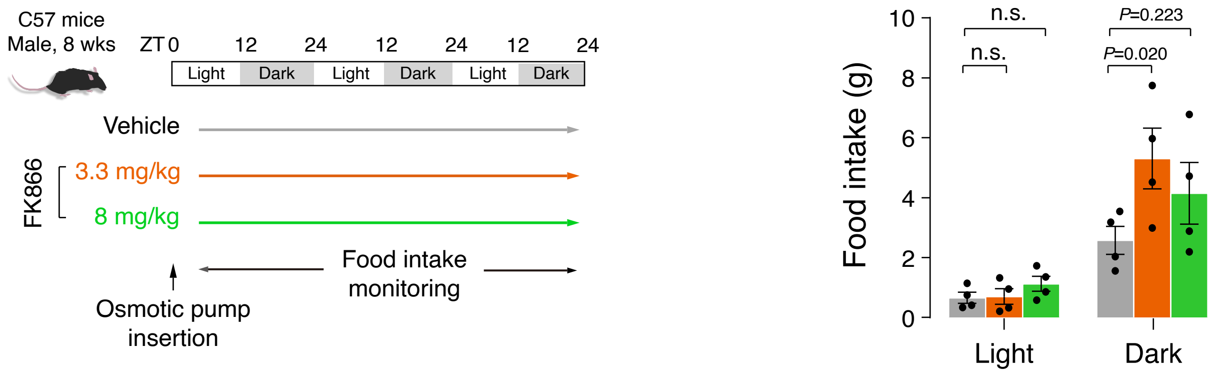

Supplementary Figure 3. Systemic NAMPT inhibition increases nighttime food intake

Mice were infused with either vehicle or FK866 (3.3 and 8 mg/kg, respectively) intraperitoneally using an osmotic pumps for 3 days. Daytime and nighttime food intakes were measured, and the average values are presented (n = 4 mice). Two-way ANOVA followed by Fisher's LSD test. Two independent replicates were performed. ZT: zeitgeber time, n.s.: not significant. Data are presented as mean ± SEM.

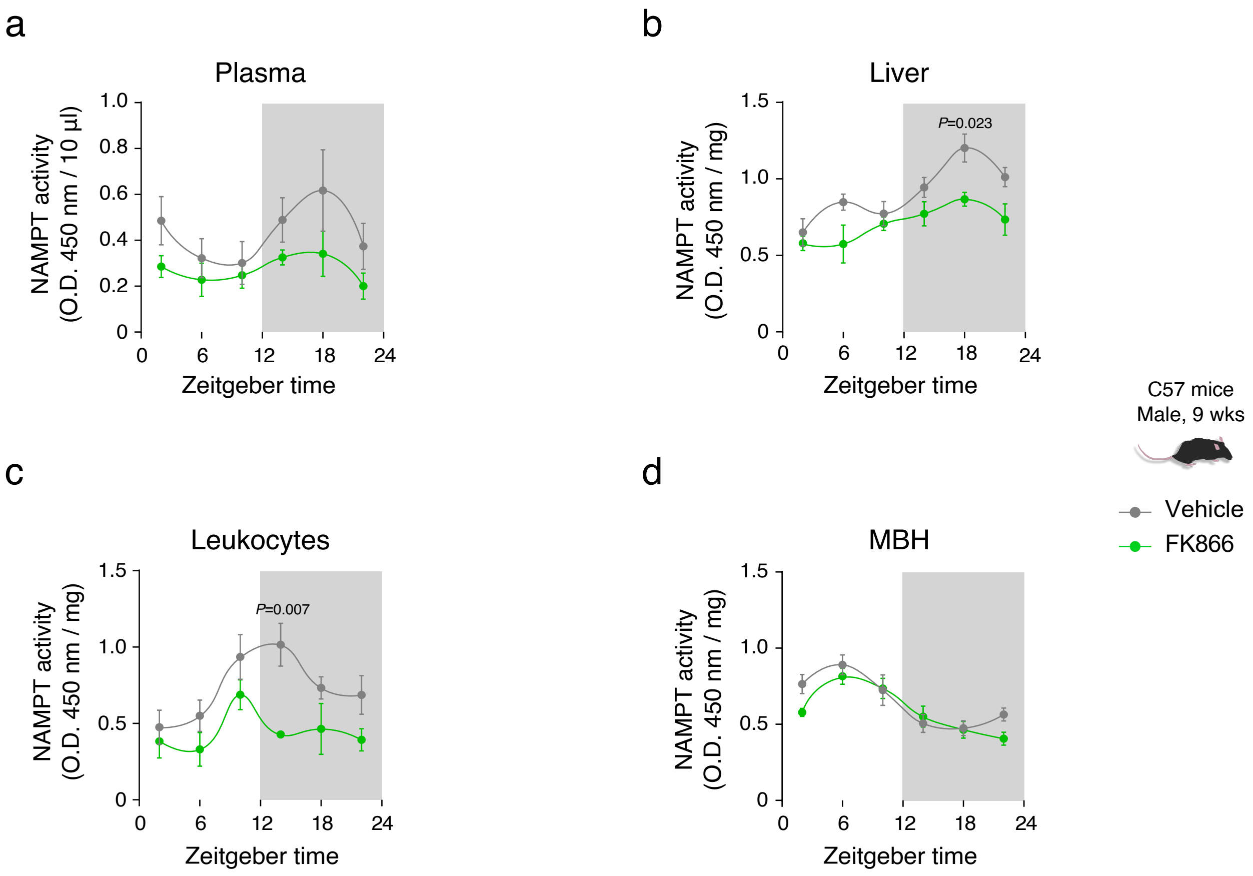

Supplementary Figure 4. Effect of systemic FK866 infusion on circadian fluctuations in NAMPT activity in the plasma, liver, leukocytes and MBH

Mice were infused with either vehicle or FK866 (8 mg/kg) via intraperitoneal-implanted osmotic pumps for 3 days and sacrificed every 4 h throughout a 24 h-circadian cycle (Leukocytes, *n* = 3 pooled samples, one sample was pooled from 3 mice; other groups, *n* = 5 mice). Repeated measures ANOVA followed by Fisher's LSD test. Two independent replicates were performed. ZT: zeitgeber times, MBH: mediobasal hypothalamus. All data are presented as mean ± SEM.

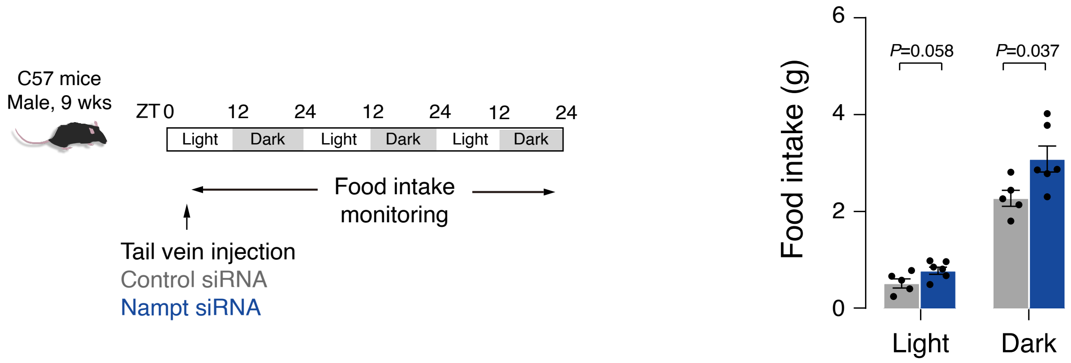

Supplementary Figure 5. Systemic *Nampt* knockdown increases nighttime food intake

Mice were injected with control or Nampt siRNA through tail vein. Daytime and nighttime food intakes were measured and the average values are presented (siControl, n = 5; siNampt, n = 6 mice). Unpaired t-test (two sided). Two independent replicates were performed. n.s.: not significant. Data are presented as mean ± SEM.

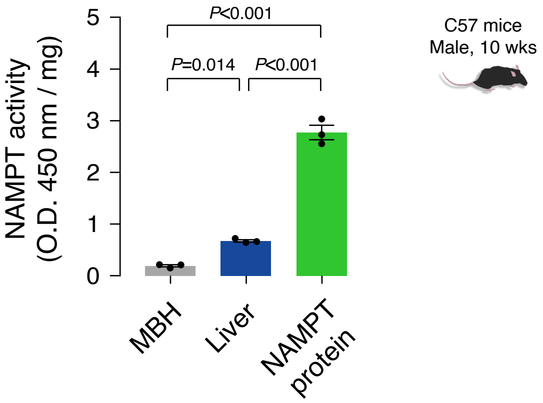

Supplementary Figure 6. Confirmation of NAMPT enzyme activity of NAMPT protein

The NAMPT activity of NAMPT protein was compared with endogenous NAMPT activity in the mediobasal hypothalamus (MBH) and liver. Tissue was collected at zeitgeber time (ZT) 6 in mice (*n* = 3 mice). One-way ANOVA followed by Fisher's LSD test. Data are presented as mean ± SEM.

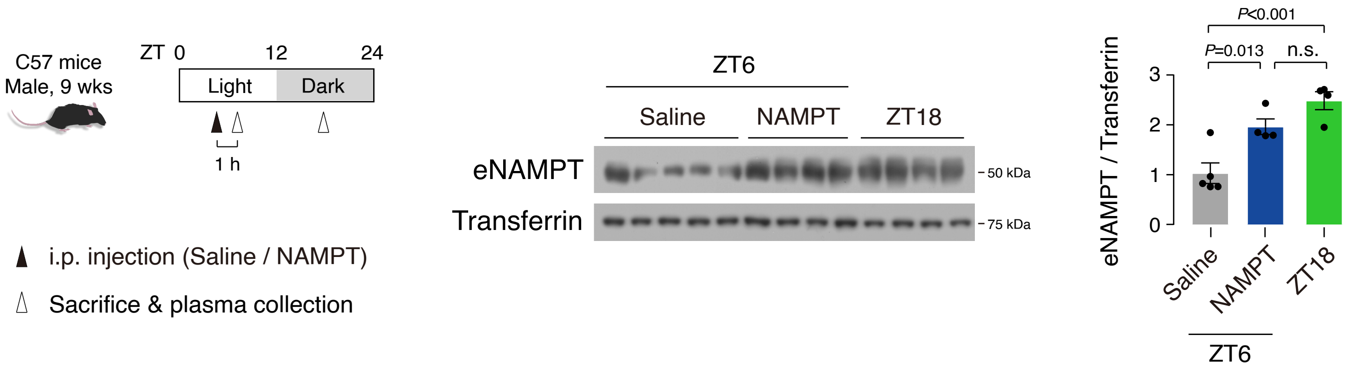

Supplementary Figure 7. Intraperitoneal injection of NAMPT protein increases plasma eNAMPT levels

Plasma was collected from mice at 1 h after intraperitoneal injection of saline or NAMPT protein (0.3 mg/kg) at ZT6. Plasma was also collected at ZT18 for comparison of eNAMPT levels (ZT6 Saline, n = 5; ZT6 NAMPT and ZT18, n = 4 mice). Plasma eNAMPT levels were determined using immunoblotting and normalized by the amounts of plasma transferrin. One-way ANOVA followed by Fisher's LSD test. Two independent replicates were performed. ZT: zeitgeber time, n.s.: not significant. Data are presented as mean ± SEM.

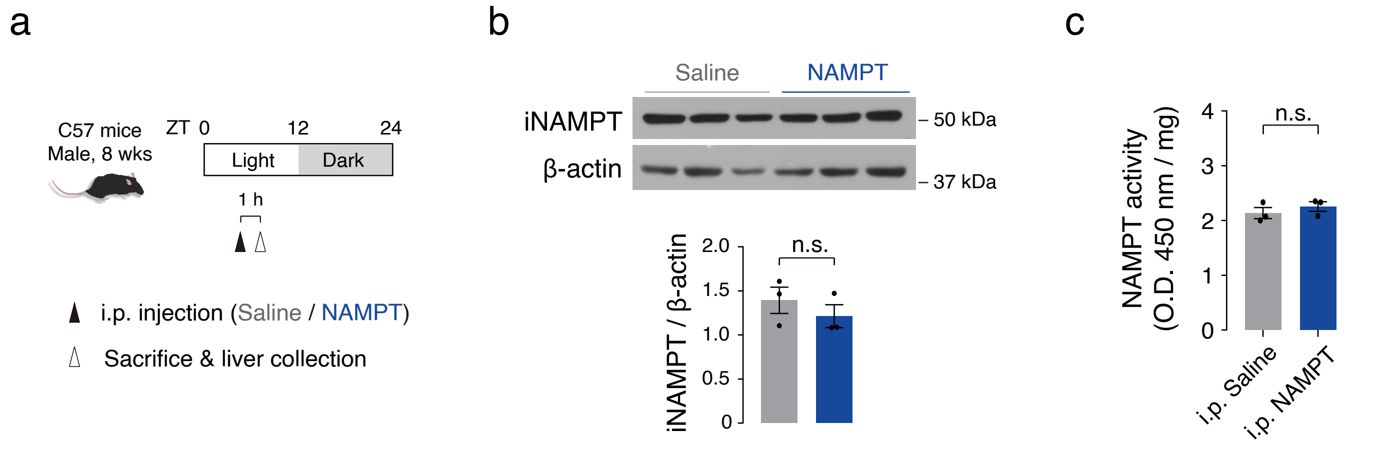

Supplementary Figure 8. Effects of intraperitoneal NAMPT administration on liver iNAMPT expression and enzyme activity

a-c Mice received intraperitoneal (i.p.) injection of saline or NAMPT protein (0.3 mg/kg) at ZT5 and liver was collected at ZT6 (*n* = 3 mice). **a** Schematic diagram of experiment. **b** Liver iNAMPT immunoblotting. **c** NAMPT enzyme activity. Unparied *t*-test (two-sided). ZT: zeitgeber time, n.s.: not significant. Two independent replicates were performed. Data are presented as mean ± SEM.

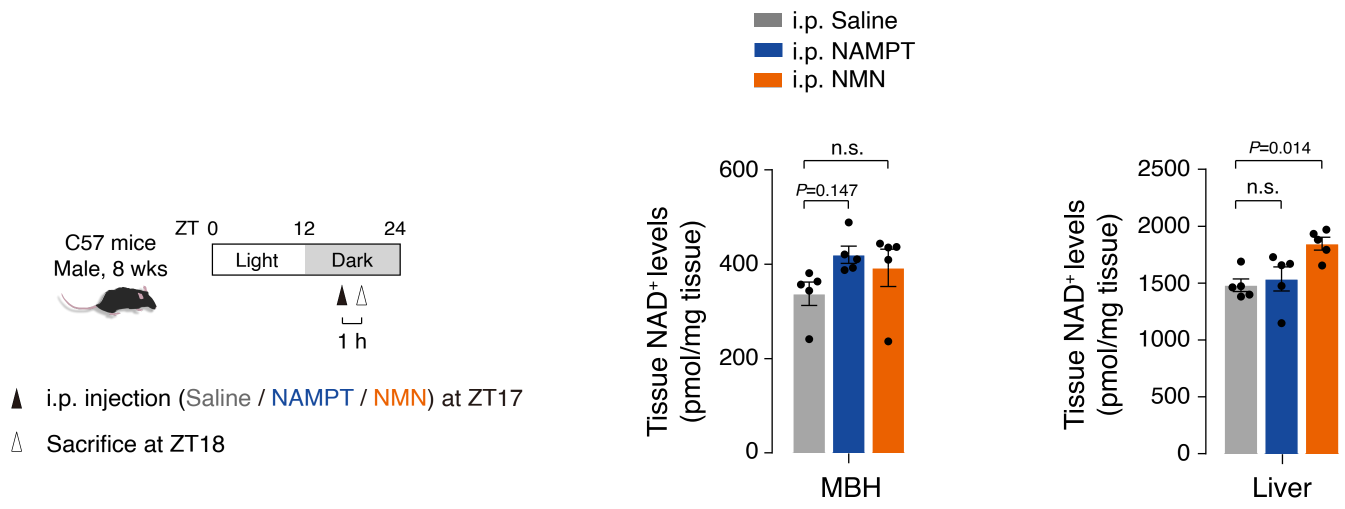

Supplementary Figure 9. Effects of NAMPT/NMN administration during the dark period on the NAD$^+$ levels in the liver and MBH

Mice received intraperitoneal (i.p.) injection of saline, NAMPT protein (0.3 mg/kg) or NMN (120 mg/kg) at ZT17. They were sacrificed at ZT18. Mediobasal hypothalamus (MBH) and liver were collected for the NAD$^+$ measurement (n = 5 mice). One-way ANOVA followed by Fisher's LSD test. Two independent replicates were performed. ZT: zeitgerber time. n.s.: not significant. Data are presented as mean ± SEM.

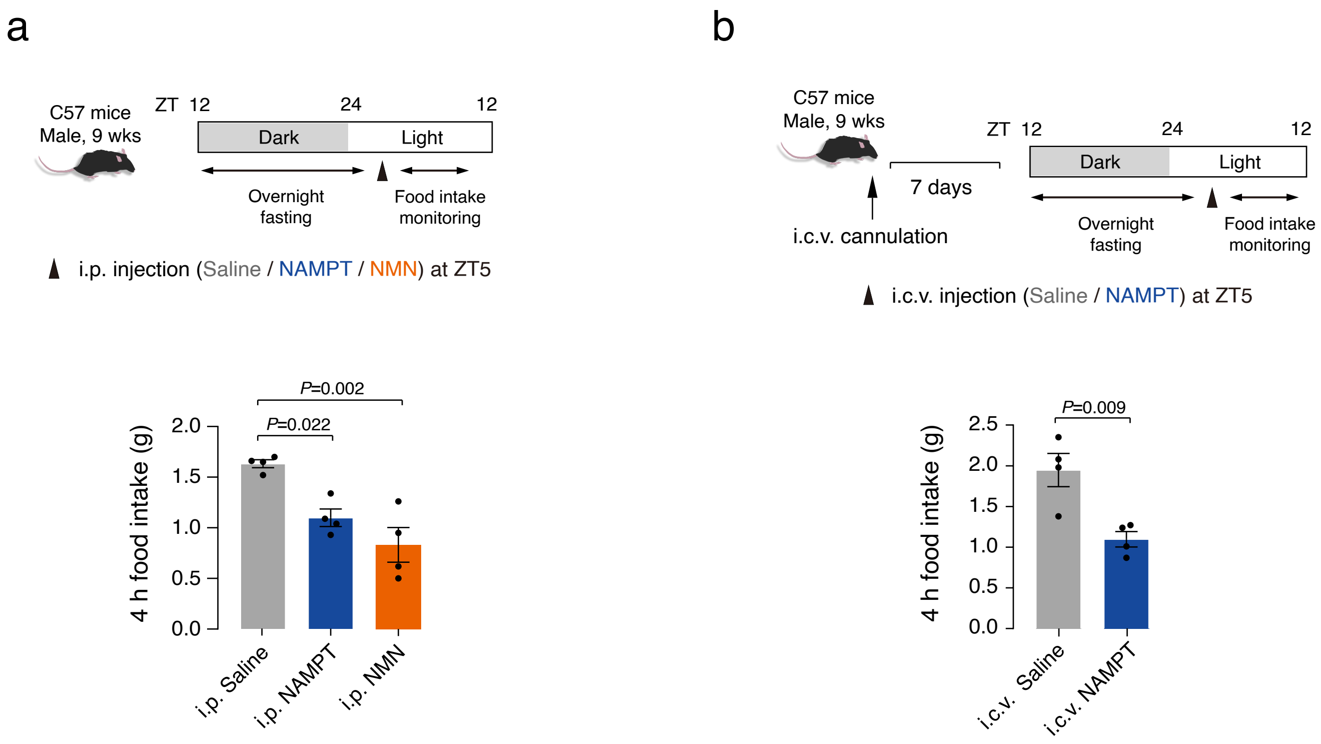

Supplementary Figure 10. Intraperitoneal and intracerebroventricular administration of NAMPT protein suppresses fasting-induced hyperphagia

a Following overnight-fasting, mice had intraperitoneal (i.p.) injection of saline, NAMPT (0.3 mg/kg) or NMN (120 mg/kg). Food intake was monitored for 4 h after the injections (n = 4 mice). One-way ANOVA followed by Fisher's LSD test. Two independent replicates were performed. **b** Mice received intracerebroventricular (i.c.v.) administration of saline or NAMPT (0.1 μg). Food intake was monitored for 4 h after the injections (n = 4 mice). Unpaired t-test (two-sided). Two independent replicates were performed. ZT: zeitgeber time.

Data are presented as mean ± SEM.

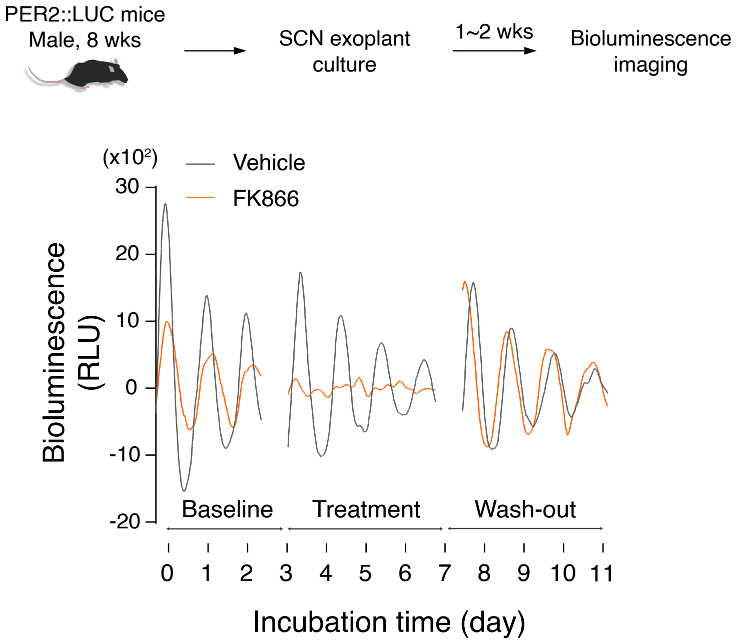

Supplementary Figure 11. Preliminary study of the effect of FK866 treatment on the SCN clock activity

Two organotypic slices including suprachiasmatic nucleus (SCN) were obtained from PER2:LUC mice and maintained in culture medium. One slice was cultured in the medium containing FK866 (20 nM) for the treatment period and the other slice was cultured in the medium containing vehicle. The SCN clock activity was determined by bioluminescence monitoring of PER2 luciferase activity. The deconvolution data of bioluminescence are presented.

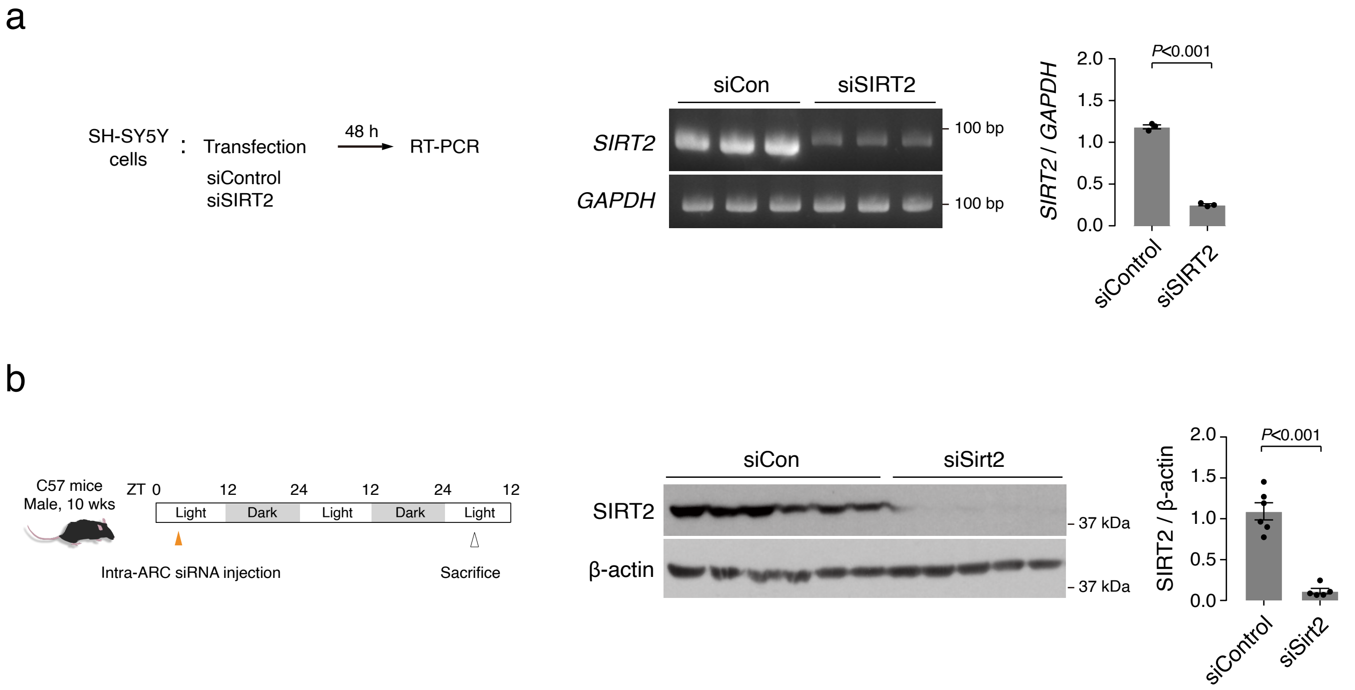

Supplementary Figure 12. Confirmation of successful SIRT2 depletion following *siSirt2* treatment

a Representative examples of successful SIRT2 depletion in SH-SY5Y cells. Cells were transfected with small inhibitory RNA specific to human Sirt2 (siSirt2) or non-targeting control siRNA (siControl) (50 nM each). Forty-eight hours later, RNA was extracted to check for successful knockdown of *SIRT2* (n = 3 wells). Unparied t-test (two-sided). **b** Representative examples of successful SIRT2 deletion in mice hypothalamus. siSirt2 or siControl (0.5 nmol each side) was injected into the bilateral arcuate nucleus (ARC) of mice. The mediobasal hypothalamus was collected on day 2 after siRNA injection for SIRT2 immunoblotting (siControl, n = 6; siSirt2, n = 5 mice). Unparied t-test (two-sided). All data are presented as mean ± SEM.

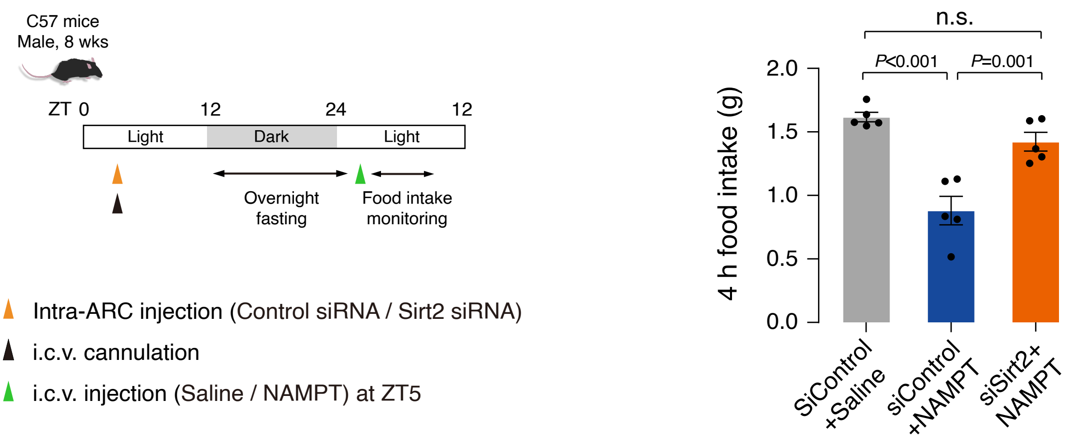

Supplementary Figure 13. Effect of intracerebroventricular administration of NAMPT on food intake in mice with hypothalamic Sirt2 depletion

Mice had bilateral intra-arcuate nucleus (ARC) injection of siControl or siSirt2 (0.5 nmol each side). On the second day after siRNA injections, mice received intracerebroventricular (i.c.v.) administration of saline or NAMPT (0.1 µg) following an overnight fasting. Food intake was monitored for 4 h after i.c.v. injection (n = 5 mice). One-way ANOVA followed by Fisher's LSD test. Two independent replicates were performed. ZT: zeitgeber time. n.s.: not significant. Data are presented as mean ± SEM.

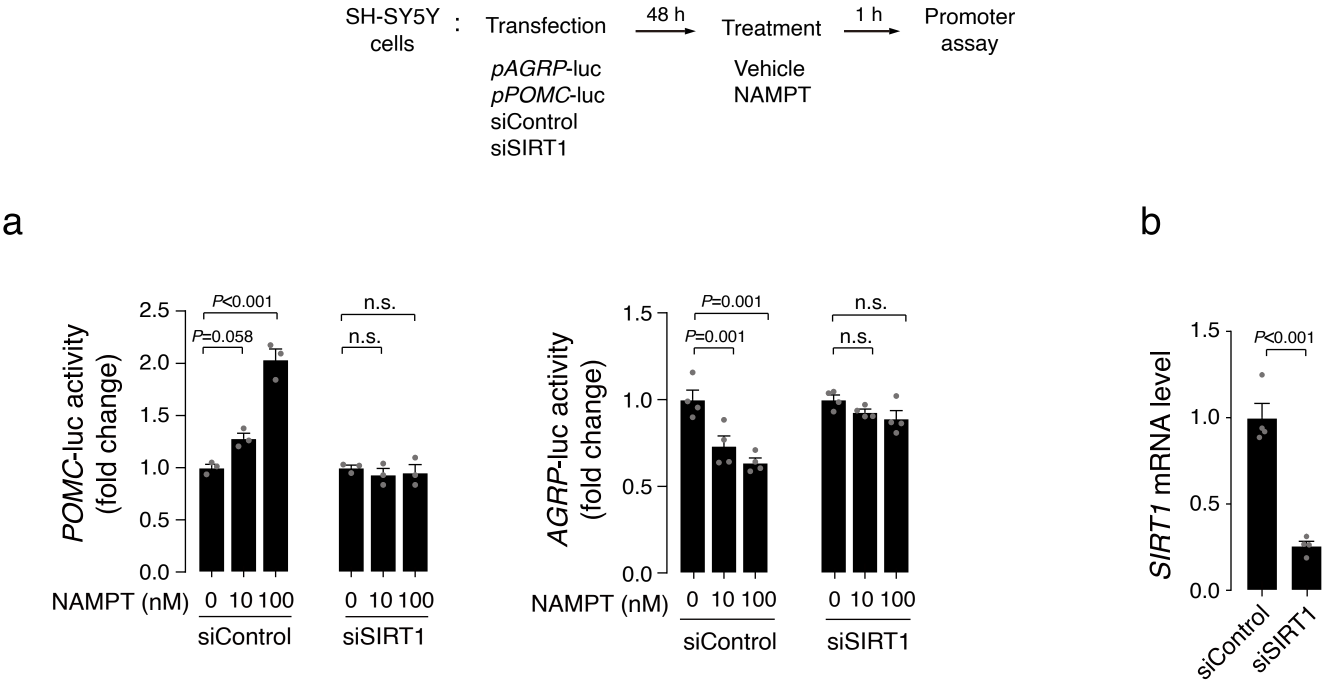

Supplementary Figure 14. Effects of *SIRT1* knockdown on NAMPT regulation of *POMC* and *AGRP* promoter activity

a SH-SY5Y cells were transfected with either siSIRT1 or siControl. 48 h later, cells were treated with vehicle or NAMPT for 1 h before harvest for promoter assay (*POMC*-luc activity, $n = 3$; *AGRP*-luc activity, $n = 4$ cell wells). One-way ANOVA followed by Fisher's LSD test. Three independent replicates were performed. n.s.: not significant.

b Representative examples of successful *SIRT1* knockdown in cells treated with either siSIRT1 or siControl ($n = 4$ cell wells). Unparied t-test (two-sided). Three independent replicates were performed.

All data are presented as mean ± SEM.

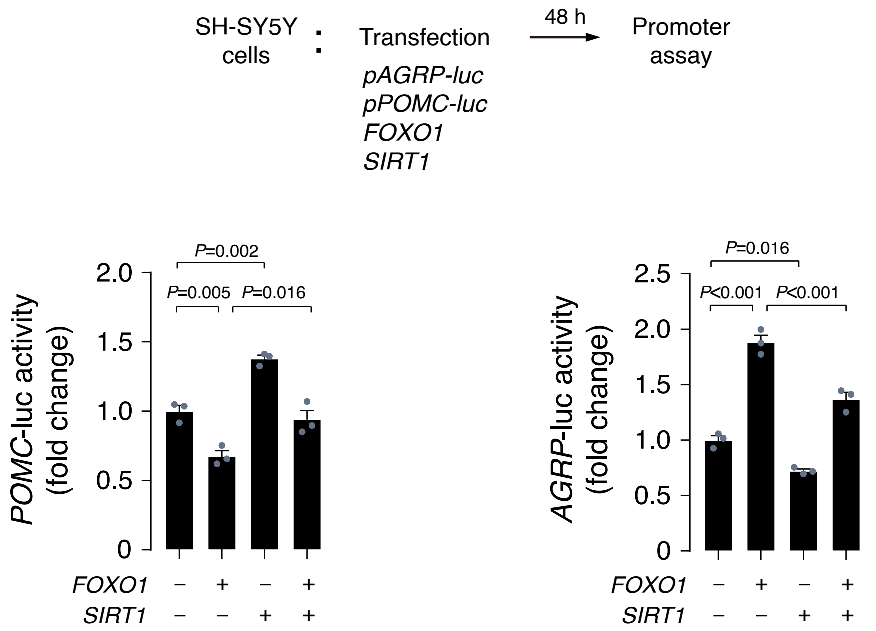

Supplementary Figure 15. The effect of *SIRT1* overexpression on *FOXO1* overexpression-induced changes in the *POMC* and *AGRP* promoter activities

POMC or *AGRP* promoter activity was assayed in SH-SY5Y cells overexpressing *SIRT1* and *FOXO1* alone or together (n = 3 cell wells). One-way ANOVA followed by Fisher's LSD test. Three independent replicates were performed. Data are presented as mean ± SEM.

a

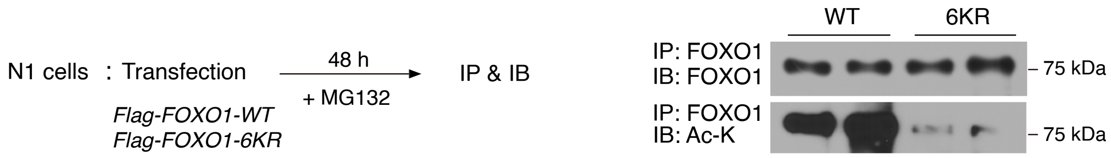

b

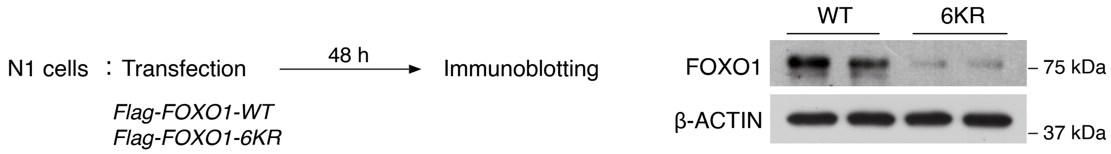

Supplementary Figure 16. Effect of FOXO1-6KR expression on FOXO1 acetylation and total protein expression

a N1 cells were transfected with the plasmids expressing *FOXO1-WT* or *FOXO1-6KR*. After transfection, cells were maintained with or without protease inhibitor MG132 treatment (10 μM) for 48 h before harvest (*n* = 2 cell wells). Cellular protein extracts were subjected to immunoprecipitation (IP) with FOXO1 antibody and then immunoblotting (IB) with FOXO1 and acetylated lysine (Ac-K). **b** N1 cells were transfected with the plasmids expressing FOXO1-WT or FOXO1-6KR. At 48 h after transfection, cellular protein was extracted for FOXO1 immunoblotting (*n* = 2 cell wells). **a**, **b** Two independent replicates were performed.

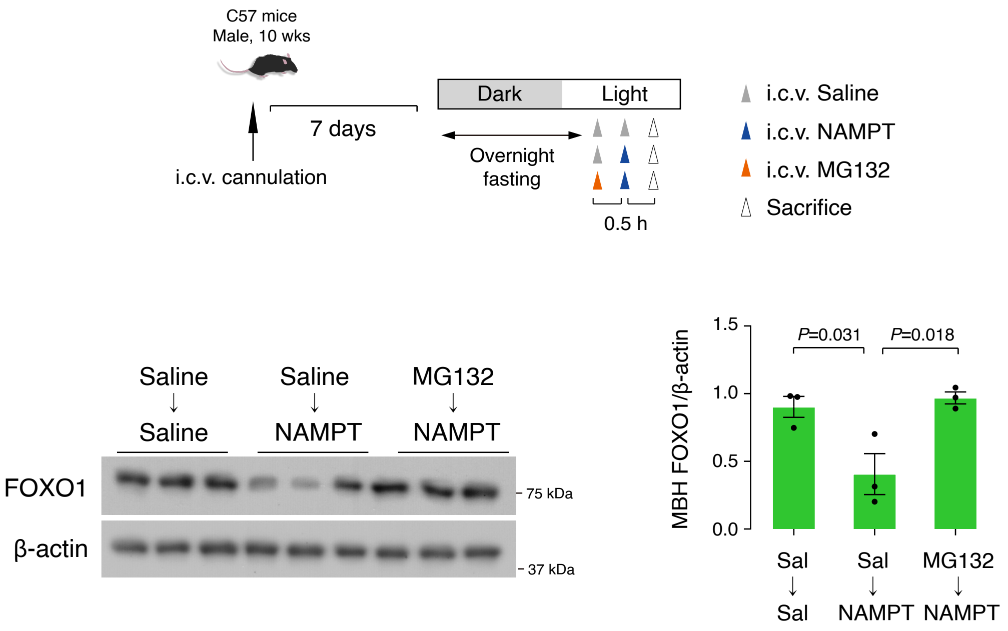

Supplementary Figure 17. Prior administration of protease inhibitor prevents NAMPT-induced hypothalamic FOXO1 degradation

Following overnight fasting, mice received serial intracerebroventricular (i.c.v.) injections of Saline→Saline, Saline→NAMPT (0.1 µg) or protease inhibitor MG132 (1 µg)→NAMPT (0.1 µg) at an interval of 30 min. The mediobasal hypothalamus (MBH) was collected 30 min after the second injections for FOXO1 immunoblotting (n = 3 mice). One-way ANOVA followed by Fisher's LSD test. Two independent replicates were performed. Data are presented as mean ± SEM.

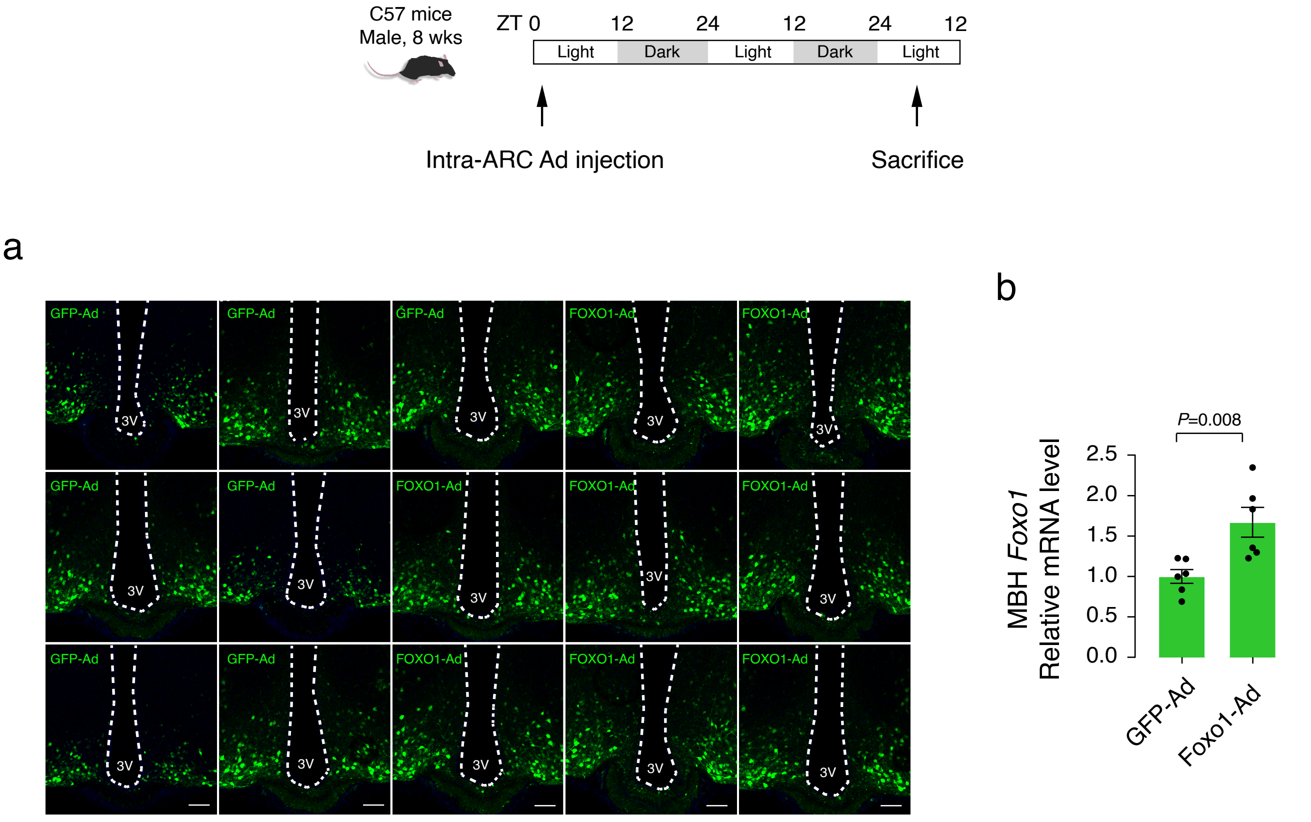

Supplementary Figure 18. Confirmation of successful viral infection

a Representative images of GFP immunostaining showing successful viral expression in the hypothalamus. Mice were injected with either Foxo1-GFP adenovirus (Foxo1-Ad) or GFP-adenovirus (GFP-Ad) into the bilaterally into the arcuate nucleus (ARC). Scale bars indicate 50 µm. 3V: The third ventricle.

b Representative examples of qPCR analysis showing successful *Foxo1* overexpression in the hypothalamus. Hypothalamic Foxo1 mRNA expression were increased in mice with Foxo1-Ad injection (n = 6 mice). Unparied t-test (two-sided). Data are presented as mean ± SEM.

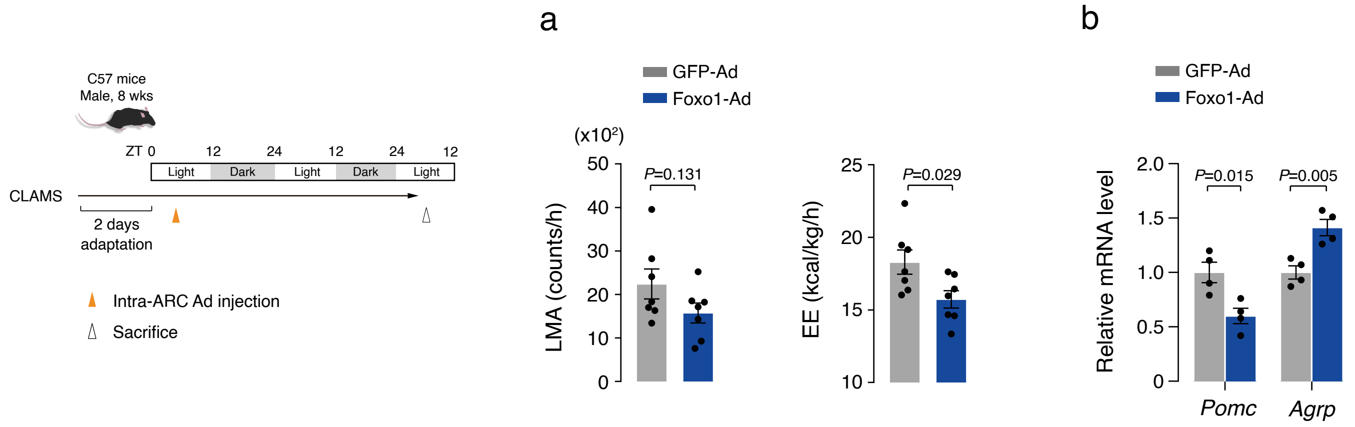

Supplementary Figure 19. Effect of ARC *Foxo1* overexpression on LMA, EE and *Pomc*/*Agrp* expression

a, **b** Mice were injected with GFP-Ad or Foxo1-Ad bilaterally into the arcuate nucleus (ARC).

a Locomotor activity (LMA) and energy expenditure (EE) were monitored after virus injection until sacrificed (n = 7 mice). **b** The mediobasal hypothalamus was collected for the measurement of *Pomc* and *Agrp* transcript levels using qPCR analysis (n = 4 mice). **a**, **b** Unparied t-test (two-sided). Two independent replicates were performed. Data are presented as mean ± SEM.

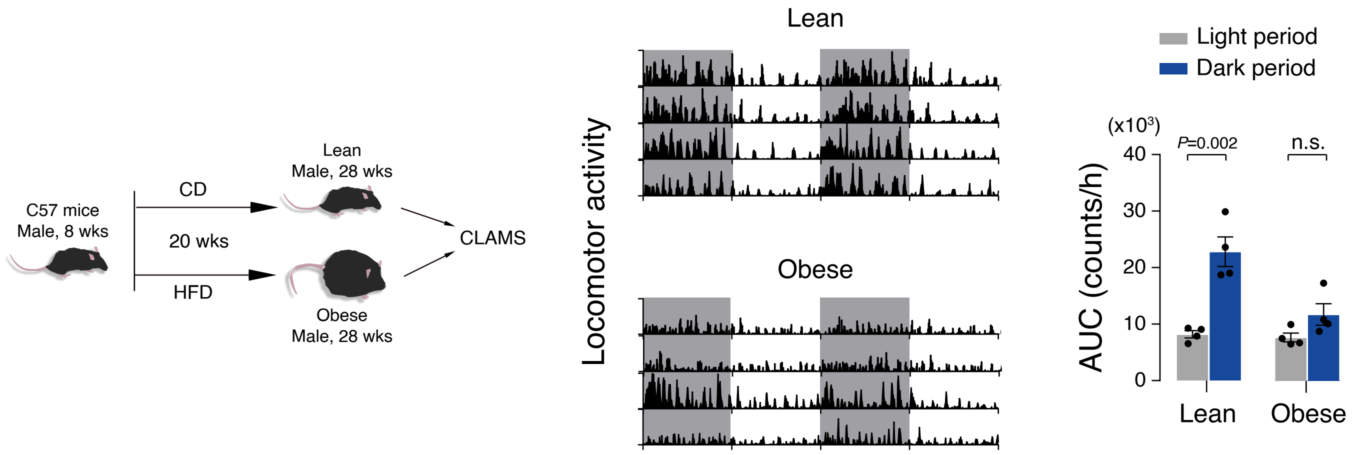

Supplementary Figure 20. Blunted circadian rhythm of locomotor activity in obese mice

Mice were fed a chow diet (CD) or an high fat diet (HFD, 60% fat) for 20 weeks. Locomotor activity was monitored in continuous lab animal monitoring system (CLAMS) cages in freely-fed conditions under normal light-dark cycle before being sacrificed (n = 4 mice). Unparied t-test (two-sided). n.s.: not significant. Data are presented as mean ± SEM.

Supplementary Table 1. Circadian rhythm parameters of hypothalamic NAD^+ levels, blood eNAMPT and tissue iNAMPT expression levels in C57 normal mice.

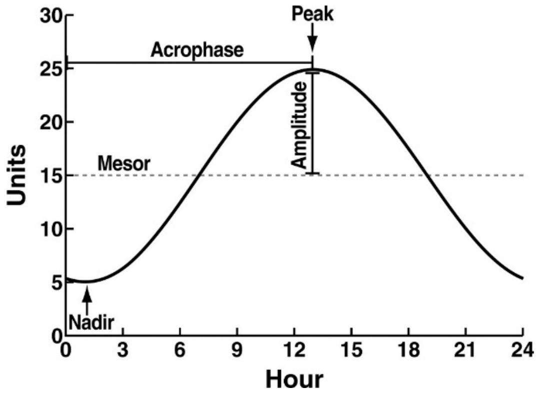

Organs/Cells	Factors	Cosinor analysis			JTK-cycle
		Mesor	Amplitude	Acrophase (h)	*P* value
MBH	NAD^+	310.7 ± 6.4	57.3 ± 9.7	17.6 ± 2.5	0.001
	NAD^+ (CD)	320.4 ± 9.3	71.8 ± 13.3	19.3 ± 2.6	0.001
	iNAMPT	1.46 ± 0.07	0.8 ± 0.1	2.8 ± 1.8	0.001
ARC	NAD^+	279.1 ± 3.2	75.4 ± 11.6	17.89 ± 2.3	0.001
Plasma	eNAMPT	0.7 ± 0.06	0.4 ± 0.08	20.9 ± 2.9	0.001
Leukocytes	*Nampt*	2.6 ± 0.3	2.7 ± 0.4	12.1 ± 2.2	0.005
Liver	iNAMPT	0.9 ± 0.07	0.470 ± 0.1	17.8 ± 3.2	0.030
Adipose tissue	iNAMPT	1.1 ± 0.08	0.3 ± 0.1	8.1 ± 5.2	0.213

C57 young mice (8 weeks old) were kept in 12 h light dark cycle and ad libitum fed condition unless indicated and sacrificed every 4 h during the 24 h period (n = 2~5 per group). JTK-CYCLE algorithm ('MetaCycle' package of R software, version 4.1.1) was used to detect circadian rhythmicity and circadian rhythm parameters were assessed using the cosinor package of R software (version 4.1.3; R Foundation for Statistical Computing, Vienna, Austria) ($P < 0.05$). Circadian rhythms parameters were obtained using cosinor analysis. Data are presented as estimate ± standard error. MBH: mediobasal hypothalamus. ARC: hypothalamic arcuate nucleus. CD: continuous darkness. Statistical significance was defined at $P < 0.05$.

Supplementary Table 2. Circadian rhythm parameters of hypothalamic SIRT1, SIRT2 and FOXO1 expression levels in C57 normal mice.

Organs	Factors	Cosinor analysis			JTK-cycle
		**Mesor**	**Amplitude**	**Acrophase (h)**	***P* value**
MBH	SIRT1	0.40 ± 0.04	0.11 ± 0.06	8.04 ± 7.93	0.058
	SIRT2	0.75 ± 0.03	0.07 ± 0.04	11.88 ± 8.73	0.251
	FOXO1	1.57 ± 0.09	1.04 ± 0.14	6.82 ± 1.82	0.001

C57 young mice were kept in 12 h light dark cycle and ad libitum fed condition and sacrificed every 4 h during the 24 h period (n = 2~5 per group). JTK-CYCLE algorithm ('MetaCycle' package of R software, version 4.1.1) was used to detect circadian rhythmicity and circadian rhythm parameters were assessed using the cosinor package of R software (version 4.1.3; R Foundation for Statistical Computing, Vienna, Austria) ($P < 0.05$). Circadian rhythms parameters were obtained using cosinor analysis. MBH: mediobasal hypothalamus. Data are presented as estimate ± standard error.

Supplementary Table 3. Circadian rhythm parameters of hypothalamic NAD^+, FOXO1, plasma eNAMPT and liver NAMPT mRNA levels in lean and obese mice.

Factors	Groups	Cosinor analysis			JTK cycle
		Mesor	Amplitude	Acrophase (h)	*P* value
MBH NAD^+	Lean mice	374.9 ± 14.5	60.5 ± 20.5	17.8 ± 4.9	0.015
	Obese mice	320.6 ± 7.8^a	28.8 ± 11.15	17.7 ± 5.6	0.286
Plasma eNAMPT	Lean mice	10.9 ± 0.6	2.9 ± 0.8	20.3 ± 4.0	0.003
	Obese mice	12.2 ± 0.3	3.7 ± 0.5	3.4 ± 1.9^c	0.001
Liver *Nampt*	Lean mice	1.7 ± 0.1	0.7 ± 0.2	12.0 ± 3.4	0.011
	Obese mice	1.7 ± 0.08^b	0.06 ± 0.11^c	15.1 ± 27.7	0.999
MBH FOXO1	Lean mice	1.2 ± 0.09	0.8 ± 0.1	6.1 ± 2.3	0.022
	Obese mice	1.3 ± 0.04	0.2 ± 0.06^c	17.6 ± 3.6^c	0.001

Obese mice were fed a high fat diet for 20 weeks before being sacrifice and lean mice were fed chow diet for the same period. The mice were maintained in 12 h light dark cycle and ad libitum fed condition and sacrificed every 4 hours during the 24 h period (n = 2~5). JTK-CYCLE algorithm ('MetaCycle' package of R software, version 4.1.1) was used to detect circadian rhythmicity and circadian rhythm parameters were assessed using the cosinor package of R software (version 4.1.3; R Foundation for Statistical Computing, Vienna, Austria) ($P < 0.05$). Circadian rhythms parameters were obtained using cosinor analysis and their differences between lean and obese group were compared using wald test to compare amplitude and acrophase between lean and obese mice while Student's *t*-test was used for the comparison of mesor. Data are presented as estimate ± standard error. $^aP < 0.05$, $^bP < 0.01$, $^cP < 0.005$ vs lean controls. n.s.: not significant. MBH: mediobasal hypothalamus.

Supplementary Table 4. Characteristics of human subjects

Subject Number	Gender	BMI (kg/m^2)	Total-C (mg/dL)	LDL-C (mg/dL)	Fasting glucose (mmol/L)	Fasting insulin (μU/mL)	Fasting leptin (ng/mL)	HOMA -IR
1	M	20.0	144.0	85.0	5.0	9.1	0.95	2.0
2	M	20.6	197.0	128.0	4.8	8.7	1.15	1.9
3	M	22.9	147.0	94.0	4.5	7.7	0.77	1.5
4	M	22.9	161.0	101.0	5.5	10.1	3.15	2.5
5	M	22.9	161.0	96.0	4.8	14.8	4.09	3.2
6	F	21.6	168.0	94.0	4.49	5.1	13.58	1.12
7	F	20.0	177.0	80.0	4.49	3.7	9.68	0.82
8	M	27.3	191.0	137.0	5.8	11.9	3.59	3.1
9	M	27.6	166.0	102.0	5.2	9.8	2.11	2.3
10	M	27.5	190.0	127.0	4.9	11.0	7.17	2.4
11	F	28.1	180.0	131.0	4.51	9.8	17.42	2.09
12	M	33.3	190.0	130.0	4.6	41.4	9.76	8.5
13	M	33.3	196.0	133.0	5.6	13.9	3.51	3.5
14	F	33.8	128.0	84.0	4.59	9.2	28.17	2.26

BMI: body mass index, Total-C: total cholesterol, LDL-C: low density lipoprotein-cholesterol, HOMA-IR: homeostasis model assessment for insulin resistance

Supplementary Table 5. Primer sequences used in qPCR

Gene	Forward (5' → 3')	Reverse (5' → 3')
Agrp	ACAACTGCAGACCGAGCA	GACGCGGAGAACGAGACT
β-actin	GGCTGTATTCCCCTCCATCG	CCAGTTGGTAACAATGCCATGT
Baml1	TTGGCCAGAGCTTGTTTGAC	CGCAGTGTCCGAGGAAGATA
Clock	AGAACTTGGCATTGAAGAGTCTC	GTCAGACCCAGAATCTTGGC
Cry1	CACTGGTTCCGAAAGGGACTC	CTGAAGCAAAAATCGCCACCT
Foxo1	CCCAGGCCGGAGTTTAACC	GTTGCTCATAAAGTCGGTGCT
Gapdh	AGGTCGGTGTGAACGGATTTG	TGTAGACCATGTAGTTGAGGTCA
Nampt	ATCCAGGAGGCCAAAGAAGT	ATCGGGAGATGACCATCGTA
Nkx2.1	AAAGCACACGACTCCGTTCT	CTCCATGCCCACTTTCTTGT
Npy	GGACTGACCCTCGCTCTA	TCGCAGAGCGGAGTAGTA
Ox2r	GAGGATTCCCTCTCTCGTCG	GGTGTAGGTATTCCCTCCACA
Per2	CTGCTGGCAGAGAGGGTACA	CATCCACAGCCTGGAACAAG
Pomc	CAGGTCCTGGAGTCCGAC	CATGAAGCCACCGTAACG
SIRT1	TGTGTCATAGGTTAGGTGGTGA	AGCCAATTCTTTTTGTGTTCGTG
SIRT2	CACGCAGAACATAGATACCCTG	CAGTGTGATGTGTAGAAGGTGC
GAPDH	CTGGGCTACACTGAGCACC	AAGTGGTCGTTGAGGGCAATG
