# Supplementary material for: Circulating blood eNAMPT drives the circadian rhythms in locomotor activity and energy expenditure
Source: Nat Commun. 2023 Apr 8;14:1994. doi: 10.1038/s41467-023-37517-6 (PMC10082796; doi:10.1038/s41467-023-37517-6)
Supplement: Supplementary file 3 — Reporting Summary [file 41467_2023_37517_MOESM3_ESM.pdf]

nature portfolio

Corresponding author(s): Min-Seon Kim

Last updated by author(s): Jan 12, 2023

Reporting Summary

Nature Portfolio wishes to improve the reproducibility of the work that we publish. This form provides structure for consistency and transparency in reporting. For further information on Nature Portfolio policies, see our Editorial Policies and the Editorial Policy Checklist.

Statistics

For all statistical analyses, confirm that the following items are present in the figure legend, table legend, main text, or Methods section.

n/a	Confirmed	
☐	☒	The exact sample size (*n*) for each experimental group/condition, given as a discrete number and unit of measurement
☐	☒	A statement on whether measurements were taken from distinct samples or whether the same sample was measured repeatedly
☐	☒	The statistical test(s) used AND whether they are one- or two-sided<br>*Only common tests should be described solely by name; describe more complex techniques in the Methods section.*
☒	☐	A description of all covariates tested
☐	☒	A description of any assumptions or corrections, such as tests of normality and adjustment for multiple comparisons
☐	☒	A full description of the statistical parameters including central tendency (e.g. means) or other basic estimates (e.g. regression coefficient) AND variation (e.g. standard deviation) or associated estimates of uncertainty (e.g. confidence intervals)
☐	☒	For null hypothesis testing, the test statistic (e.g. *F*, *t*, *r*) with confidence intervals, effect sizes, degrees of freedom and *P* value noted<br>*Give P values as exact values whenever suitable.*
☒	☐	For Bayesian analysis, information on the choice of priors and Markov chain Monte Carlo settings
☒	☐	For hierarchical and complex designs, identification of the appropriate level for tests and full reporting of outcomes
☒	☐	Estimates of effect sizes (e.g. Cohen's *d*, Pearson's *r*), indicating how they were calculated

Our web collection on statistics for biologists contains articles on many of the points above.

Software and code

Policy information about availability of computer code

Data collection	Comprehensive Lab Animal Monitoring System (CLAMS, Columbus Instruments),<br>LSM 700 confocal microscope (Carl Zeiss),<br>Real-time PCR analysis (Quantstudio5, Applied biosystems),<br>Dish-type wheeled luminometer (AB-2550 Kronos-Dio, ATTO),<br>Luminometer (PerkinElmer, Monza)
Data analysis	Image analysis:<br>ZEN microscope software (version 2.1 blue edition, Carl Zeiss),<br>Image J (Version 1.52p)<br><br>Data analysis:<br>SPSS version 24 (IBM analytics),<br>JTK-CYCLE algorithm 'MetaCycle' package of R software (version 4.1.1),<br>Cosinor package of R software (version 4.1.3),<br>Graphpad Prism (version 7)<br>Microsoft Excel 2016,<br>The background-subtracted bioluminescence profiles were analyzed using the Cosinor analysis software (http://www.circadian.org).

For manuscripts utilizing custom algorithms or software that are central to the research but not yet described in published literature, software must be made available to editors and reviewers. We strongly encourage code deposition in a community repository (e.g. GitHub). See the Nature Portfolio guidelines for submitting code & software for further information.

Data

Policy information about availability of data

All manuscripts must include a data availability statement. This statement should provide the following information, where applicable:

- Accession codes, unique identifiers, or web links for publicly available datasets
- A description of any restrictions on data availability
- For clinical datasets or third party data, please ensure that the statement adheres to our policy

Source data for figures are provided with the paper.

Human research participants

Policy information about studies involving human research participants and Sex and Gender in Research.

Reporting on sex and gender	14 healthy Koreans (10 males, 4 females)
Population characteristics	The human participants who underwent testing were with no medical history of chronic illness or malignancy. The average age of total 14 subject was 23.1 + or - 1.8 years. Seven subjects (5 males, 2 females) were BMI < 23 kg/m2 and 7 subjects (5 males, 2 females) were BMI > 27 kg/m2, according to the definition of obesity from the International Diabetes Federation-Western Pacific Region. The characteristics of the study population are detailed in Supplementary Table 4.
Recruitment	Participants were recruited through local website advertising.
Ethics oversight	Institutional Review Board of Asan Medical Center (IRB approval number: 2008-0314, approved data: August 11th, 2008)

Note that full information on the approval of the study protocol must also be provided in the manuscript.

Field-specific reporting

Please select the one below that is the best fit for your research. If you are not sure, read the appropriate sections before making your selection.

☒ Life sciences ☐ Behavioural & social sciences ☐ Ecological, evolutionary & environmental sciences

For a reference copy of the document with all sections, see nature.com/documents/nr-reporting-summary-flat.pdf

Life sciences study design

All studies must disclose on these points even when the disclosure is negative.

Sample size	No statistical method was used to predetermine sample size in animal experiments. Cell numbers of relative experiments in this study were determined based on our experience and previous studies in this field.
Data exclusions	The animal data with failed virus and siRNA injection were excluded for data analysis.<br>Undetermined RT-qPCR data were excluded for data analysis.
Replication	All experiments were repeated at least two times and data were presented when replicates were successful.
Randomization	For i.p. or i.c.v. iARC injection experiments, mice were selected according to their body weight to match the average body weights between groups. Control and experimental samples were always processed in parallel to control covariates.
Blinding	Investigators were not blinded in conducting animal experiments or collecting samples for analysis. Where possible, researchers were blinded during data analysis.

Reporting for specific materials, systems and methods

We require information from authors about some types of materials, experimental systems and methods used in many studies. Here, indicate whether each material, system or method listed is relevant to your study. If you are not sure if a list item applies to your research, read the appropriate section before selecting a response.

Materials & experimental systems

n/a	Involved in the study
	x Antibodies
	x Eukaryotic cell lines
x	Palaeontology and archaeology
	x Animals and other organisms
x	Clinical data
x	Dual use research of concern

Methods

n/a	Involved in the study
x	ChIP-seq
x	Flow cytometry
x	MRI-based neuroimaging

Antibodies

Antibodies used

Anti-NAMPT, mouse monoclonal (Adipogen, Catalog #:AG-20A-0034, RRID:AB_2490117, 1:1500)
Anti-FOXO1, rabbit monoclonal (Cell signaling, Catalog #:2880s, RRID:AB_2106495, 1:1000)
Anti-SIRT1, mouse monoclonal (Santa Cruz, Catalog #:sc-74465, RRID:AB_1129462, 1:1000)
Anti-SIRT2, rabbit monoclonal (Cell signaling, Catalog #:12650, RRID:AB_2716762, 1:1000)
Anti-Flag, mouse monoclonal (Sigma, Catalog #:F3165, RRID:AB_259529, 1ug)
Anti-Acetylated lysine, rabbit polyclonal (Cell signaling, Catalog #:9441, RRID:AB_331805, 1:1000)
Anti-c-Fos, rabbit polyclonal (Synaptic systems, Catalog #:226 003, RRID:AB_2231974, 1:1000)
Anti-β-actin, mouse monoclonal (Santa Cruz, Catalog #:sc-47778, RRID:AB_626632, 1:1000)
Anti-β-endorphin, rabbit polyclonal (Phoenix, Catalog #:H02233, RRID:AB_2314007, 1:1000)
Anti-GFP, goat polyclonal (Abcam, Catalog #: ab6673, RRID:AB_305643, 1:1000)
Anti-rabbit secondary antibody, donkey polyclonal, Alexa-Flour 488-conjugated (Invitrogen, Cat#: A21206; RRID: AB_141708)
Anti-rabbit secondary antibody, donkey polyclonal, Alexa-Flour 555-conjugated (Invitrogen, Cat#: A31572; RRID: AB_162543)

Validation

Antibody validation information can be found on manufacturers' website.
Anti-NAMPT (mouse), https://adipogen.com/ag-20a-0034-anti-nampt-visfatin-pbef-mab-omni379.html
Anti-FOXO1 (rabbit), https://www.cellsignal.com/products/primary-antibodies/foxo1-c29h4-rabbit-mab/2880
Anti-SIRT1 (mouse), https://www.scbt.com/p/sirt1-antibody-b-7
Anti-SIRT2 (rabbit), https://www.cellsignal.com/products/primary-antibodies/sirt2-d4o5o-rabbit-mab/12650
Anti-Flag (mouse), https://www.sigmaaldrich.com/KR/ko/product/sigma/f3165
Anti-Acetylated lysine (rabbit), https://www.cellsignal.com/products/primary-antibodies/acetylated-lysine-antibody/9441
Anti-c-Fos (rabbit), https://www.citeab.com/antibodies/509572-226-003-c-fos-rabbit-polyclonal-affinity-purifieda
Anti-β-actin (mouse), https://www.scbt.com/p/beta-actin-antibody-c4
Anti-β-ENDORPHIN (rabbit), https://www.phoenixpeptide.com/products/view/Antibodies/H-022-33
Anti-GFP (goat), https://www.abcam.com/gfp-antibody-ab6673.html

Eukaryotic cell lines

Policy information about cell lines and Sex and Gender in Research

Cell line source(s)

SH-SY5Y human neuroblastoma cell line were obtained from the ATCC (Cat#:CRL-2266).
N1 embryonic mouse hypothalamus cell line were obtained from the CEDARLANE (Cat#:CLU101).

Authentication

SH-SY5Y cells and N1 cells were from commercial sources and thus not subsequently validated.

Mycoplasma contamination

SH-SY5Y cells and N1 cells were not contaminated with mycoplasma.

Commonly misidentified lines
(See ICLAC register)

None.

Animals and other research organisms

Policy information about studies involving animals; ARRIVE guidelines recommended for reporting animal research, and Sex and Gender in Research

Laboratory animals

C57BL/6 male mice were purchased from Orient Bio (Seongnam, korea).
Mice with tdTomato-labelled POMC neurons were generated by mating POMC-Cre mice (provided by Dr. Joel K. Elmquist, University of Texas Southwestern Medical Center) with mice carrying the reporter Rosa26-loxP-STOP-loxP-tdTomato allele (Jackson Laboratory, 007909). NPY-hrGFP mice (Jackson Laboratory, 006417) were used for the purpose of AgRP neuron labeling. PER2:LUC knock-in mice used for the SCN clock study were provided by Dr. Joshep S. Takahashi (University of Texas Southwestern Medical Center).

Wild animals

The study did not involve wild animals.

Reporting on sex

All studies were conducted in male mice.

Field-collected samples	The study did not include field-collected samples.
Ethics oversight	All animal procedures were approved by the Asan Institutional Animal Care and Use Committee of the Institute for Life Science under the project license 2012-11-024.

Note that full information on the approval of the study protocol must also be provided in the manuscript.
